# THE GROWING DEMAND FOR AGE-FRIENDLY CARE AND UNDERSERVED OLDER ADULTS IN THE HEALTH CARE SYSTEM

**DOI:** 10.1093/geroni/igae098.4299

**Published:** 2024-12-31

**Authors:** Katy Terveer

**Affiliations:** Age Wave, Orinda, California, United States

## Abstract

This session will present on the challenges and opportunities to re-think our health care system based on the latest findings from “Meeting the Growing Demand for Age-Friendly Care: Health Care at a Crossroads,” a study released by Age Wave and The John A. Hartford Foundation. We will explore the experiences and pain points of older adults, particularly those underserved, in today’s health care system based on a recently conducted survey with over 2,500 older adults ages 65+. We will identify key findings for those who are the least satisfied with their health care, namely, those with low financial resources, women, rural older adults, and Black and Hispanic older adults. We’ll summarize the key recommendations from the report for transforming health care systems to support longer healthspans and greater well-being as we age.

